# 35 Years of Partnership to Advance Cardiovascular Health and Well-Being in American Indian Communities: The Strong Heart Study and Strong Heart Family Study

**DOI:** 10.5888/pcd22.250216

**Published:** 2025-07-10

**Authors:** Amanda M. Fretts, Jessica A. Reese, Ying Zhang

**Affiliations:** 1 University of Washington, Department of Epidemiology, Seattle; 2 University of Oklahoma Health Sciences Center, Oklahoma City

The value of longitudinal studies in population health research cannot be overstated. Longitudinal studies, particularly prospective cohort studies, allow scientists to better understand associations of social, behavioral, clinical, and environmental factors with disease risk or progression over time — sometimes decades (1). These studies provide key sources of information needed to inform public health policy and interventions intended to maximize health outcomes for all people in the US.

The Strong Heart Study (SHS) and Strong Heart Family Study (SHFS) are 2 cohort studies funded by the National Heart, Lung, and Blood Institute (NHLBI) that have been ongoing since 1989 and 2001, respectively (2,3). The studies comprise 12 American Indian communities in Arizona, Oklahoma, North Dakota, and South Dakota. At the inception of the SHS, little was known about cardiovascular diseases in rural and American Indian communities because all the other large cohort studies that focused on cardiovascular diseases (eg, Cardiovascular Health Study, Framingham Heart Study, Coronary Artery Risk Development in Young Adults study) did not include American Indian people. The SHS was developed to better understand risk factors for cardiovascular diseases among American Indians in particular (2,4). In 2001, the study was expanded to include family members of participants from the original cohort. That study, called the SHFS, was designed to better understand the heritability of cardiovascular diseases (3). Over the past 35 years, scientists and participating communities have developed a better understanding of the major risk factors for cardiovascular diseases and worked together to develop, implement, and evaluate several interventions intended to improve cardiovascular health (5–10). These interventions have had meaningful impacts on the cardiovascular health of the participating communities, including a 47% reduction in urinary arsenic levels and increased self-reported use of arsenic-safe drinking and cooking water (5), significant reductions in systolic blood pressure, low-density lipoprotein cholesterol, and left ventricular mass (8), and increased awareness of the effect of physical activity and cholesterol on risk of cardiovascular diseases (10). Furthermore, data from the first 25 years of the SHS indicate a decrease in the incidence of cardiovascular diseases among both men and women and a decrease in deaths from cardiovascular diseases among men (11).

As part of the SHS and SHFS, participants completed in-person examinations every 3 to 14 years (2,3). Over time, the focus of the examinations shifted from collection of data on traditional risk factors for cardiovascular diseases (eg, smoking, diabetes, hypertension) to include novel risk factors of clinical, community, and public health importance (eg, environmental heavy metal exposures, metabolomics, food environment, resilience). These additional data collection components were driven by an evolving scientific landscape and changes in community interests. For instance, in the late 1990s, there was a strong interest in genetic factors that may contribute to the risk of cardiovascular diseases, and the inclusion of family members of original SHS participants enabled detailed genetic analyses (3). More recently, community members expressed concerns about potential heavy metals in groundwater and soil, and scientists have assessed associations of blood or urinary arsenic, uranium, and lead with several health outcomes by using data collected over the past 35 years (12–14).

This week *Preventing Chronic Disease* features 7 articles from the SHS and SHFS. The articles are an example of the wide breath of hypotheses that can be tested with available data from the well-designed cohort studies and illustrate the importance of examining multifaceted social, behavioral, clinical, and environmental factors that may affect cardiovascular health. The articles deepen our knowledge of cardiovascular health in populations historically underrepresented in research and highlight 3 overarching themes: 1) the benefit of forging partnerships and sharing knowledge, 2) the importance of collecting data on multilevel and multidimensional risk factors using rigorous scientific methods, and 3) the value of stored biospecimens in longitudinal studies. Taken together, the collection features a successful 35-year partnership of scientists and tribes committed to promote optimal health outcomes in rural communities.

## Forging Partnerships and Sharing Knowledge

The SHS (n = 3,500) and SHFS (n = 2,753) are the largest and longest ongoing cohort studies of cardiovascular diseases and risk factors among American Indians in the US, and a major strength of the SHS and SHFS is the involvement of multiple tribes across 4 states (4). The SHS and SHFS involve collaborations across participating tribes, academic institutions, and the NHLBI. These collaborations are bidirectional. As described by Howard et al, the tribes and academic partners work together at every stage of the study (15). This includes development of research goals based on community interests, strengths and priorities; creation or adaptation of data collection instruments; implementation of research protocols; interpretation of study results; dissemination of findings to tribal leadership, citizens, and local health-serving organizations; and exploring new research directions and next steps.

Some articles featured in this collection are direct products of community-initiated hypotheses. For instance, many participating tribes stress the importance of holistic health and the interconnections of physical, spiritual, emotional, and mental well-being. Because of community interest and concerns regarding the potential link between depression and cardiovascular diseases, Santori et al assessed the association of depressive symptoms with hypertension risk in the SHFS (16). Results indicate that participants who reported depressive symptoms at baseline had 54% higher odds of developing hypertension compared with participants with no depressive symptoms (16). Similarly, community concerns regarding exposure to heavy metals from groundwater and soil set the foundation for a deep dive into environmental determinants of health in the SHS and the SHFS. In their article, Patterson et al reported positive associations of urinary uranium levels with hypertension (17). Findings from these reports underscore the importance of aligning research goals with community interests and priorities to accelerate scientific discoveries of local public health importance.

## Importance of Collection of Multilevel and Multidimensional Risk Factors by Using Rigorous Scientific Methods

The SHS and SHFS include comprehensive in-person examinations that use instruments with known validity and reliability (2,3). The data from these examinations enable scientists to conduct robust data analyses and adequately address confounding (1). Furthermore, the collection of data on a wide range of topics across several dimensions of health — sociodemographic, behavioral, clinical, and household factors; family history of chronic diseases; and the local environment — enables both scientists and the participating tribes to understand the complex interplay of multilevel factors on health outcomes.

A wide range of data are collected at the SHS and SHFS in-person examinations and through ongoing surveillance (ie, annual telephone calls and medical record reviews to ascertain major clinical events). Additionally, with permission from the Indian Health Service Institutional Review Boards, Tribal Research Review Boards, and study participants, the SHS and SHFS query several national health repositories and data sources to augment the type and precision of data available in the studies. The article by Fabsitz et al showcases an example of the use of combining data from the SHS and the Indian Health Service National Data Warehouse (18). The SHS and SHFS also use other sources, including the Centers for Medicare and Medicaid Services, state cancer registries, state vital records, and the National Death Index. Use of multiple data sources for event ascertainment ensures data quality and reliability of research findings and maximizes utility of the study.

## Value of Stored Biospecimens in Longitudinal Studies

The COVID-19 pandemic taught us that it is not possible to anticipate what health will look like in the coming years. In this collection, 3 articles report on biomarkers that were not measured at the time of the in-person examinations and laboratory assessments but rather used stored specimens collected at past SHS and SHFS examinations (17,19,20). Freezing and storing biospecimens for future use is efficient for achieving medical advances in a timely fashion because it allows scientists to examine the relationship of exposures in the distance past (as measured using stored blood or urine) with current health outcomes. Without these historical biospecimens, scientists would need to design a study, collect biomarkers, and wait years for enough participants to develop the outcome to assess if the biomarker is relevant to health. The use of stored biospecimens in research often leads to new interventions and therapies to improve health decades before it would be possible had these biospecimens not been available. For instance, at the time of the 2001–2003 examination, the use of mass spectrometry to measure the lipidome was largely unknown in large cohort studies. However, Chen et al were able to use stored samples from the 2001–2003 SHFS examination to measure lipidomics in 2017 (20).

Although the use of stored biospecimens in research studies is a time-, labor-, and cost-effective way to learn from the past to help ensure a healthier future for current and future generations, use of these samples also presents challenges. Strong academic–community partnerships based on trust, transparency, and ongoing dialogue are critical to ensure best practices for safeguarding biospecimens. There must be an upfront and clear understanding of where biospecimens will be stored, how long they will be stored, and how they will be used in advance of the study. In the SHS and SHFS, participants were asked permission to save biospecimens for future use (ie, opt-in or opt-out) and provided with a clear indication of what types of analyses the specimens may be used for. The Strong Heart Study Observational Study Monitoring Board, the Indian Health Service Institutional Review Boards, and Tribal Research Review Boards must review and approve all studies that use any SHS or SHFS data, including studies that propose to use stored biospecimens. Additionally, in the SHS and SHFS, every biological sample maintains a spiritual connection to those who donate it. The academic institutions and tribes have worked closely to determine the best way to honor these sacred samples, including blessings of the laboratories and biospecimens by tribal leaders. If at any time a study participant decides that they no longer wish to have their biospecimens used in research, the academic institutions and tribes work together to honor the tribe and participant’s request in a culturally respectful way.

## Future Directions

Looking ahead to the next 35 years of the SHS and SHFS, we hope to expand the study in several ways. Both scientists and tribes agree that early prevention of cardiovascular risk factors is critical to optimize health outcomes. The article by Reese et al, which focused solely on American Indian adolescents, demonstrated a high prevalence of cardiovascular risk factors (19). We hope to expand the study to adolescents and young adults to better understand early life risk factors for cardiovascular diseases and how best to maximize health throughout the life course. Additionally, we hope to continue to prioritize community capacity building. As part of the current SHS and SHFS cycle, the NHLBI provided funds for a handful of community members and organizations in the SHS and SHFS communities to design and lead pilot projects to fill scientific, policy, or community health needs to improve health status based on their interests or goals. These grants helped to center community voices and strengthen local research capacity. The SHS and SHFS are also working to design a user-friendly web interface that the tribes can use to better use SHS and SHFS data and leverage results to support internal programming. To encourage efficient data harmonization and spur research collaborations that support clinical and health policy decisions, scientists are also mapping SHS data to existing National Institutes of Health Common Data Elements (CDEs) and developing SHS- and SHFS-specific CDEs (21). Finally, findings from the SHS and SHFS have informed development of several community-based cardiovascular health promotion interventions over the past 35 years (5–7), and we hope to continue this work moving forward.

The SHS and SHFS are built on decades of trust, transparency, power-sharing, mutual learning, and a shared commitment to prevent cardiovascular diseases and promote longevity among American Indians. The goals of the study have evolved over time but demonstrate the value in academic–community partnerships to define health challenges and solutions to maximize health and well-being for all.
